# Feasibility of Exceeding 20% Efficiency for Kesterite/c-Silicon Tandem Solar Cells Using an Alternative Buffer Layer: Optical and Electrical Analysis

**DOI:** 10.3390/nano14211722

**Published:** 2024-10-29

**Authors:** Naoufal Ennouhi, Safae Aazou, Abdeljalile Er-rafyg, Zakaria Laghfour, Zouheir Sekkat

**Affiliations:** 1 Department of Chemistry, Faculty of Sciences, Mohammed V University in Rabat, Rabat BP 1014, Morocco; ennouhi.naoufal@gmail.com (N.E.); s.aazou@um5r.ac.ma (S.A.); rabdeljalile@gmail.com (A.E.-r.); 2 Optics and Photonics Center, Moroccan Foundation for Advanced Science & Innovation & Research, MAScIR-UM6P, Rabat BP 10100, Morocco; zakaria.laghfour@gmail.com

**Keywords:** tandem solar cell, kesterite, c-Si, SCAPS-1D, current matching conditions

## Abstract

Tandem solar cells have the potential to be more efficient than the Shockley–Queisser limit imposed on single junction cells. In this study, optical and electrical modeling based on experimental data were used to investigate the possibility of boosting the performance of kesterite/c-Si tandem solar cells by inserting an alternative nontoxic TiO_2_ buffer layer into the kesterite top subcell. First, with SCAPS-1D simulation, we determined the data reported for the best kesterite (CZTS (Eg = 1.5 eV)) device in the experiments to be used as a simulation baseline. After obtaining metric parameters close to those reported, the influence on the optoelectronic characteristics of replacing CdS with a TiO_2_ buffer layer was studied and analyzed. Different top subcell absorbers (CZTS0.8Se0.2 (Eg = 1.4 eV), CZTS (Eg = 1.5 eV), CZTS (Eg = 1.6 eV), and CZT0.6Ge0.4S (Eg = 1.7 eV)) with different thicknesses were investigated under AM1.5 illumination. Then, to achieve current matching conditions, the c-Si bottom subcell, with an efficiency at the level of commercially available subcells (19%), was simulated using various top subcells transmitting light calculated using the transfer matrix method (TMM) for optical modeling. Adding TiO_2_ significantly enhanced the electrical and optical performance of the kesterite top subcell due to the decrease in parasitic light absorption and heterojunction interface recombination. The best tandem device with a TiO_2_ buffer layer for the top subcell with an optimum bandgap equal to 1.7 eV (CZT0.6Ge0.4S4) and a thickness of 0.8 µm achieved an efficiency of approximately 20%. These findings revealed that using a TiO_2_ buffer layer is a promising way to improve the performance of kesterite/Si tandem solar cells in the future. However, important optical and electrical breakthroughs are needed to make kesterite materials viable for tandem applications.

## 1. Introduction

Among the renewable energies, photovoltaics has been shown to be a crucial alternative for future low-cost power generation. Nevertheless, the technology faces various problems in maintaining this position, the most notable of which is the efficiency of solar cells. Current attempts show that crystalline silicon-based solar cell efficiency is reaching basic limitations based on the Shockley–Queisser limit [1]. The tandem idea allows this single junction constraint to be surpassed (multijunction). Tandem devices provide a route to greater conversion performance by maximizing solar spectrum absorption and lowering thermalization losses in the bottom subcell [2]. For these reasons, multiple efforts have been undertaken in recent years to construct novel tandem cell structures [3,4,5,6]. Due to the excellent thermal stability of silicon solar cells, which are more compatible with the deposition technique used to build the top subcell, tandem systems based on the silicon bottom subcell idea may be much more useful; in addition, some silicon heterojunctions with wide bandgaps have been used and proven to be promising for developing high efficiencies beyond the limit of homo-junction silicon solar cells [7]. The tandem structure with c-Si as bottom subcells (Eg = 1.12 eV) and a top subcell with an absorber with an Eg up to 1.7 eV theoretically yields more than 40% efficiency [8]. Perovskite-based solar cells have been proposed as an appealing material for use as wide bandgap top subcells due to their huge bandgap tunability and high efficiency exceeding 24.8% [9,10]. Several studies have investigated perovskite as a top subcell in c-Si/perovskite tandem solar cells due to recent outstanding developments in perovskite solar cell performance [6,11]. These structures yield an efficiency of 31.3% in a monolithic structure [12]. However, stability is the major challenge to success with these structures. Regarding the most recent perovskite/Si tandem device breakthroughs, there has been much interest in looking for alternative wide bandgap top subcell materials that might result in more stable and efficient tandem cells. Thin film solar cells based on CdTe and CIGS are viable options. In fact, a 16.8% efficient Cd1-xZnxTe/Si [13] and Si/CGSe tandem cell with 10% efficiency has been found [14]. Unfortunately, these materials are hampered by the toxicity and scarcity of their constituent components. Kesterite thin films could be good top subcell materials since they have good long-term stability and can be elaborated with low-cost and nontoxic materials. Moreover, the optical bandgap of kesterite varies based on its chemical composition, ranging from about 1 eV for pure CZTSe [15,16] to more than 2 eV for germanium-alloyed CZTGS [17] Additionally, the bandgap of kesterite materials can be tuned to the desired range for tandem applications using various dopants. This adaptability makes kesterite materials promising candidates for future development of tandem solar cells [18]. Kesterite devices have witnessed considerable advances in their efficiency over the course of the last several years, reaching over 11% for pure sulfide CZTS [19] and more than 13% for the sulfo-selinide ACZTSSe [20]. Researchers have recommended kesterite/c-Si as a feasible approach toward low-cost and highly efficient tandem devices for the reasons mentioned above.

Finding a suitable tunnel junction to enable smooth carrier transfer between the top and bottom subcells is one of the primary hurdles in the practical development of the CZTS/c-Si tandem device. This interface should have high infrared transparency to allow adequate light absorption in the c-Si bottom cell, strong electrical connections between top and bottom cells, and high resistance to the high temperature thermal treatments applied in top subcell fabrication [21]. The first working monolithic CZTS/c-Si tandem cell with a MoS2/FTO/ZnO intermediate layer with an efficiency of 3.5% has been reported [21]. However, the degradation of the intermediate layers’ transmittance and the elements’ diffusion into the Si bottom subcell during the high temperature treatments used to develop the top subcell are the main restrictions limiting the tandem device’s obtained performances. This problem might be solved by placing a diffusion barrier layer between the bottom cell and top cell. It has been claimed that the TiN nanolayer protects the bottom Si cell from contamination during CZTS growth and acts as an interface recombination layer [22]. Furthermore, several transition metal oxide nanolayers, such as CuO, have been employed at the back contact in CZTS solar cells, showing enhanced efficiency. These nanolayers could also be used as tunnel junctions (n++/p++) with some n-type materials in kesterite/c-Si solar cells [23]. This approach can mitigate the losses associated with the direct contact between the p-type top subcell and the n-type bottom subcell, which can lead to the formation of a reverse p-n junction.

Notwithstanding the abovementioned concerns with the intermediate connection, the power conversion efficiency (PCE) of the kesterite solar cells is too low for usage in top subcells. The primary reasons for the poor performance of the CZTS solar cell are the high recombination rate produced by either a high density of bulk and/or surface defects [24,25] and improper alignment with the CdS buffer layer [26,27]. It is critical to improve the performance of the CZTS top subcell to obtain the desired performance from the CZTS/c-Si tandem device.

Yuancai et al. [20] concluded in their analysis of the ACZTSSe/CdS solar cell that a defective and lattice-mismatched interface is created during CdS deposition, and the CdS is not the suitable buffer layer for a kesterite-based solar cell. Furthermore, numerous effective methods are being researched, such as the possibility of CdS buffer layer replacement. As nontoxic buffer layers, ZTO and ZnS have been investigated [28,29]. According to a recent study, utilizing a nontoxic TiO_2_ buffer layer considerably improves the performance of CIGS solar cells [30]. Additionally, utilizing TiO_2_ with a wide bandgap may minimize parasitic absorption in the short wavelength region and remove CdS toxicity. Since CZTS and CIGS materials have comparable characteristics, several studies have recently outlined the use of TiO_2_ as an electron transporting layer in CZTS solar cells [12]. Moreover, the results obtained from CZTS/TiO_2_ devices are comparable to the ones obtained from CZTS/CdS reference cells, suggesting that much higher performances could be obtained by applying an ALD-TiO_2_ buffer layer. Additionally, current modeling methodologies show that using TiO_2_ as an electron transport material is a good strategy to improve the performance of CZTS devices [31,32]. In addition, TiO_2_ have been used as a wide bandgap heterojunction and hole blocking layer in silicon solar cells, which prevents transport of holes while allowing transport of electrons, and maybe similar effects can be observed in kesterite solar cells [33,34]

In this paper, the use of TiO_2_ as an alternative buffer layer in kesterite solar cells and its impact on CZTS/Si tandem cells is studied via optical and electrical modeling. The top subcell kesterite is reproduced to fit the reported data from the experiments. Afterwards, adding TiO_2_ as a buffer layer for the top subcell is investigated. Moreover, both top subcell structures, kesterite/CdS and kesterite/TiO_2_, with different bandgaps and thicknesses of the absorber layer (kesterite) are simulated and analyzed to calculate the absorbed spectrum from the top subcell. The c-Si bottom subcell is simulated with various spectra of transmitted light from the top subcell. By plotting the short circuit current density (J_SC_) and the maximum power current density (J_MPP_) of the bottom and top subcells and as a function of the top subcell absorber bandgap and thickness, a current matching condition (both subcells produce similar currents) was obtained. Conclusively, the tandem solar cell characteristics are calculated under current matching conditions.

## 2. Materials and Methods

The suggested kesterite/c-Si tandem solar cell was electrically simulated using Solar Cell Capacitance Simulator (SCAPS-1D) software version 3.3.07 [35]. This software is widely used to model and simulate thin-film polycrystalline solar cells. The results from the simulations with SCAPS are very similar to those of the experiments [36]. The program is based on a numerical resolution of the semiconductor, Poisson, and carrier continuity Equations (1)–(3) to calculate the parameters of the simulated solar cells.

The Poisson equation is as follows:

(1) $$\frac{d}{dx}\left(-\varepsilon \left(x\right)\frac{d\Psi }{dx}\right)=q[p\left(x\right)-n\left(x\right)+N_{D}^{+}\left(x\right)-N_{A}^{-}\left(x\right)+p_{t}\left(x\right)-n_{t}\left(x\right)]$$

where *ε* is the dielectric constant; $N_{A}^{-}\left(x\right)$ and $N_{D}^{+}\left(x\right)$ are the acceptor and donor concentrations, respectively; Ψ is the electrostatic potential; $p_{t}\left(x\right)$ and $n_{t}\left(x\right)$ are trapped holes and electrons, respectively; $p\left(x\right)$ and $n\left(x\right)$ are the free hole and electron concentrations, respectively; and q is the electron charge.

The continuity equations for holes (2) and electrons (3) are as follows:

(2) $$\frac{1}{q}\left[\frac{dJ_{p}\left(x\right)}{dt}\right]=G_{L}\left(x\right)-R(x)$$

(3) $$\frac{1}{q}\left[\frac{dJ_{n}\left(x\right)}{dt}\right]={-G}_{L}\left(x\right)+R(x)$$

where $G_{L}\left(x\right)$ is the optical generation rate and R(x) is the net recombination rate.

Different configurations of kesterite/c-Si tandem cells can be used (2-terminal and 4-terminal solar cells). In the 4-terminal connection, the two subcells are mechanically stacked, and the outputs are collected independently using four electrodes. On the other hand, the top subcell is deposited directly on the bottom subcell in the 2-terminal configuration, and the two subcells are electrically connected in series. The 4-terminal connection is straightforward to create; however, the electrodes must be carefully chosen to minimize parasitic absorption. Whereas on the other hand, the 2T-tandem cells use only one transparent electrode, reducing parasitic absorption and manufacturing costs.

**Figure 1:**
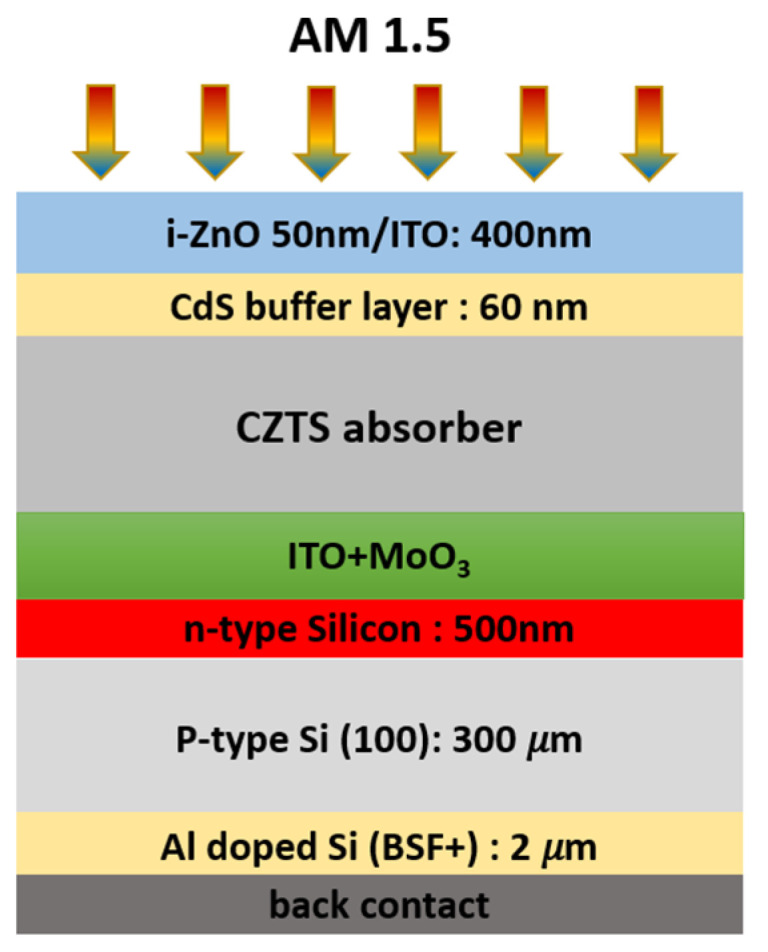
Tandem solar cell structure of CZTS/c-Si used in our simulation.

In the current study, we adopted the 2T-tandem structure depicted in Figure 1. The top subcell had ZnO (50 nm) and ITO (400 nm) transparent conducting oxide (TCO) layers, CdS (60 nm) as an n-type wide bandgap buffer layer, and kesterite as a p-type absorber. The bottom subcell contained a typical p-n junction of crystalline silicon. Table 1 summarizes the material parameters employed in our simulation. Table 2 summarizes the main defect properties used for the baseline simulation. The input parameters for each layer of CZTS, CdS, TiO_2_, and ZnO were chosen based on theories and literature values [32,37,38,39,40,41,42,43]. The complicated defect properties of kesterite solar cells are particularly sensitive to several factors, such as the experimental procedure used to prepare the various layers. In this instance, the bulk defect characteristics of kesterite materials were chosen based on Kim et al.’s work [44] and modified to match the reference cell performances at a defect density of 5.1015 cm^−3^. The CdS bulk and kesterite/CdS interface defect properties were extracted from the baseline reported in [43]. The parameters used in the simulation for the bottom c-Si solar cells were mostly from the default library of SCAPS-1D. The simulated c-Si bottom subcell exhibits an efficiency of 18.7% with a V_OC_ of 639 mV, a J_SC_ of 36.9 mA/cm^2^, and a fill factor of 79.4%, which is in good agreement with the experiment of a real solar cell reported in [45].

The practical development of monolithic kesterite/c-Si requires the use of TCOs as intermediate layers, which is now the only approach available. Ah et al. developed a CIGSe/ITO/c-Si tandem device with more than 10% efficiency [14]. According to their electrical analysis, the series resistance of the bottom subcell has the largest impact on the tandem efficiency, whereas the electrical loss owing to the tunnel junction resistance is insignificant. Furthermore, a theoretical study demonstrated that the recombination junction (RJ) has a strong effect on the fill factor of the monolithic tandem cells; however, employing a recombination junction with a lower interface gap can produce high-efficiency monolithic tandem cells without a strong effect on the RJ [46]. Based on these findings, and since the main tunneling mechanisms needed for monolithic multijunction structure simulations are not implemented in SCAPS-1D, the electrical resistance of the tunnel junction was not considered in the electrical simulation, and we simulated the top and bottom subcells independently.

**Figure 2:**
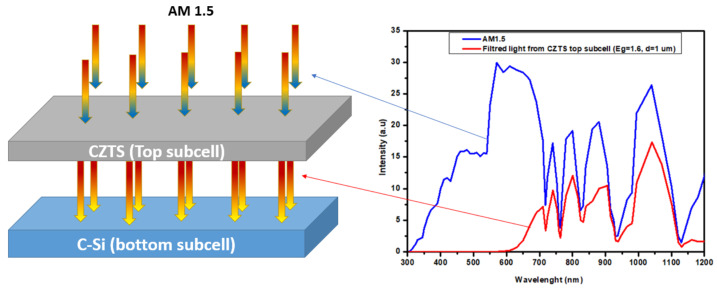
The approach used in this simulation, with the absorption of the top subcell and the transmitted light to the bottom subcell calculated and used for simulating the c-Si subcell.

The kesterite top subcell with a structure of ITO/MoO_3_/kesterite/CdS/ZnO/ITO was modeled optically to calculate the transmitted light for various bandgaps and thicknesses of the absorber layer (Figure 2). In several studies on tandem devices, the transmitted light from the top subcell was calculated using the Beer–Lambert law [37,47,48]. This method contains only absorption at the layers. Thus, no interface reflection and no interference are considered. In this work, the TMM method was used to calculate the transmittance and reflectance of the top subcell and the current matching conditions, in which both subcells producing similar J_SC_ or J_MPP_ were considered. After that, the tandem device at the current matching points was simulated to maximize the current produced in a series-connected mode.

**Figure 3:**
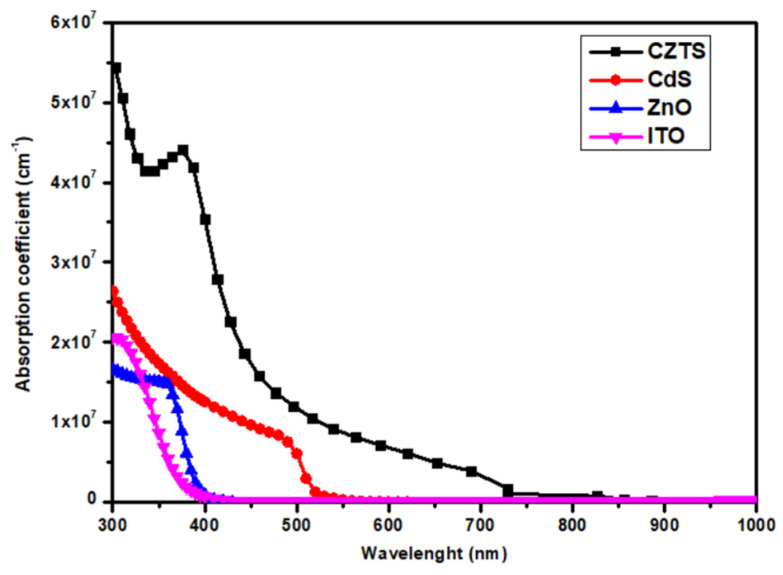
The absorption coefficient of the different materials of the baseline CZTS (Eg = 1.5 eV) solar cell.

The optical data used in the optical modeling of the top cell were extracted from experimental works. CdS, ZnO, CZTS0.8Se0.2 (Eg = 1.4 eV), and CZTS (Eg = 1.5 eV) layers are based on ellipsometry data collected by Et-taya et al. [36]. The TiO_2_ parameters are from [44], and for the kesterite thin films, CZTS (Eg = 1.6 eV), CZT0.6Ge0.4 (Eg = 1.7 eV), ITO, and MoO3 are from [43]. The absorption coefficients used in this simulation for each material were calculated from the above optical data, and the results are shown in Figure 3. Since the SCPAS optical model considers only the absorption at the layers, the reflection of the top subcell was calculated using TMM and used as an optical filter in SCAPS simulations for a more accurate calculation result.

## 3. Results

### 3.1. Validation of the Simulation Model

**Figure 4:**
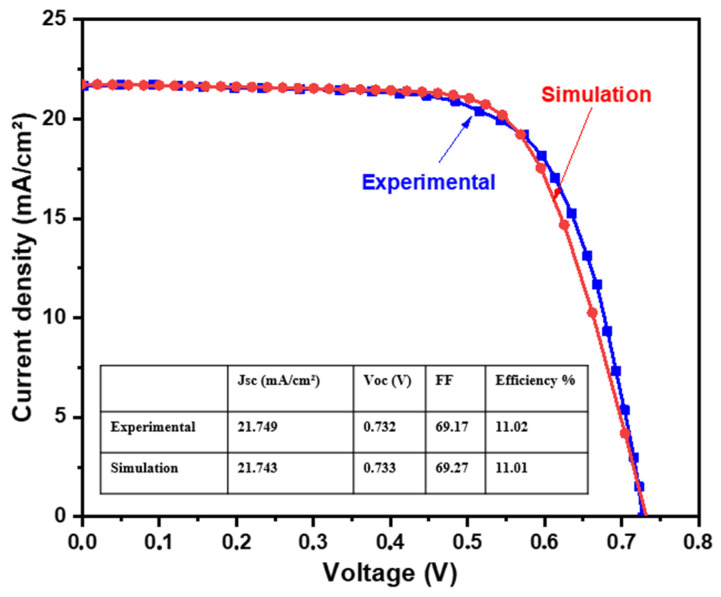
Comparison between the J-V performances of the simulated and reproduced CZTS solar cells [17].

The world record CZTS (Eg = 1.5 eV) solar cell reported by Yan et al. [19] was used to validate the input parameters and create a simulation baseline for our study. This solar cell exhibits an efficiency of 11.01%, with V_OC_ = 0.73 V, J_SC_ = 21.74 mA/cm^2^, and FF = 69.27%. A comparison of reported and simulated CZTS solar cell performance is shown in Figure 4. The calculated photovoltaic parameters for both cells are summarized in the inset table of Figure 4. The simulated device exhibits an efficiency of 11.01% with V_OC_ = 0.73 V, J_SC_ = 21.74 mA/cm^2^, and FF = 69.17%, suggesting that the difference between the experimental and simulated data is insignificant. This good match between the experiment and our numerical results confirms the accuracy of the used baseline model.

### 3.2. Improving the CZTS Performance Using an Alternative Buffer Layer

**Figure 5:**
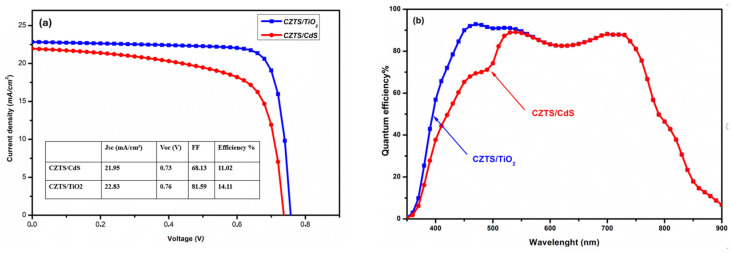
(**a**) J-V performances of the simulated CZTS solar cells with CdS (red line) and TiO_2_ (blue line) buffer layers. (**b**) Quantum efficiencies of CZTS solar cells with CdS (red line) and TiO_2_ (blue line) buffer layers.

The output characteristics achieved from the simulated CZTS/CdS solar cell are inadequate for the application of this structure as a top subcell with a silicon solar cell in a tandem design. To develop a tandem structure with acceptable efficiency, it is necessary to improve the CZTS top subcell performance. In this work, we aim to improve the performance of the kesterite device through band engineering employing TiO_2_ as a buffer layer. Based on previous research [31,49], we expect that n-type TiO_2_ creates an ideal junction with p-type kesterite and may enhance the quality of the kesterite/TiO_2_ interface, resulting in a considerable boost in CZTS device performance. To investigate only the effect of TiO_2_ on the solar cell performance, we kept all the parameters unchanged, and we replaced CdS with the suggested TiO_2_ using the input parameters listed in Table 1. Figure 5a illustrates the J-V curve of both the CZTS/CdS and CZTS/TiO_2_ structures, and their parameters are listed in the inset table. It should be mentioned that the discrepancies in the PV performance characteristics between the CZTS/CdS and CZTS/TiO_2_ devices are significant. The CZTS/TiO_2_ solar cell achieves an efficiency value of 14.11%, whereas the reference cell’s efficiency is only 11.01%. This high improvement in efficiency is due to a large increase from 68 to 81 in FF and a slight increase from 21.95 mA/cm^2^ to 22.85 mA/cm^2^ and from 0.73 V to 0.76 V in J_SC_ and V_OC_, respectively.

The quantum efficiency (QE) of CZTS/CdS and CZTS/TiO_2_ is shown in Figure 5b. It should be noted that at higher wavelengths, where the absorber dominates the absorption, both devices display almost the same QE. However, at shorter wavelengths, a strong improvement in the QE is seen in the device with the TiO_2_ buffer layer, indicating better photogenerated carrier collection and reduced recombination at the heterojunction [50,51]. Furthermore, the introduction of wide bandgap TiO_2_ contributes to the enhancement of QE by reducing parasitic absorption in this region (<500 nm).

**Figure 6:**
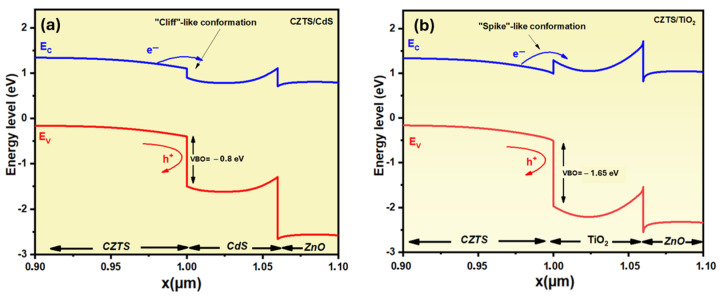
Band diagram at the heterojunction for CZTS/TiO_2_ (**a**) and CZTS/CdS (**b**).

**Figure 7:**
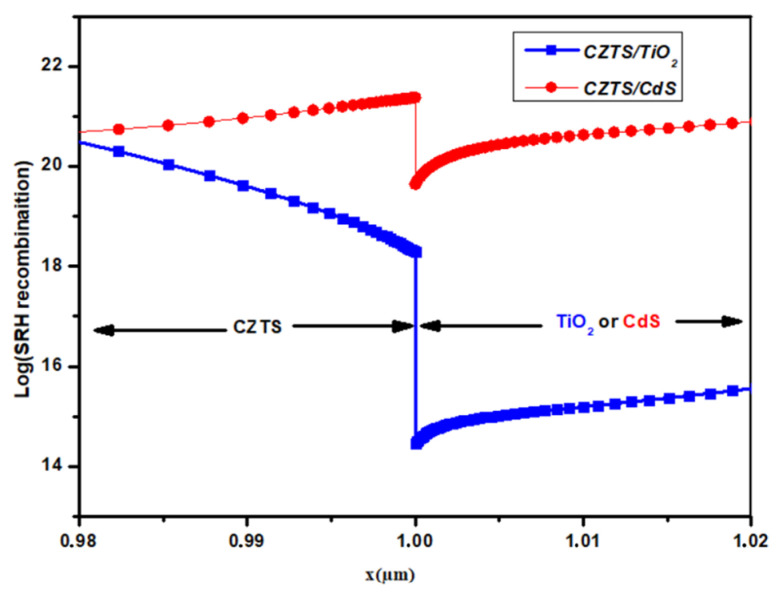
SHR recombination at the heterointerface of CZTS/CdS (red line) and CZTS/TiO_2_ (blue line) structures.

The band diagrams of CZTS/CdS and CZTS/TiO_2_ are shown in Figure 6 to address these findings. The band alignment at the heterointerface is determined by the bandgap and electron affinity of the absorber and the buffer layer; the obtained conduction band offset (CBO) in CZTS/CdS is a cliff-like configuration with value of about −0.095eV, as reported previously [52]. This cliff-like CBO is reported to have a negative effect on charge transfer between the absorber and the buffer layer and leads to a high density of carrier recombination at the interface. The CBO is changed to spike-like (Ec buffer > Ec absorber), with a value of 0.11 eV when the TiO_2_ buffer layer is employed. On the other hand, the valence band offset (VBO) of the buffer layer must be designed to prevent photogenerated holes (PGHs) at the absorber/buffer interface. In the case of the CZTS/TiO_2_ heterojunction, a VBO value of −1.67 eV is obtained, which is greater than that of CZTS/CdS (−0.8 eV). This higher VBO contributes to the improved QE and J_SC_ of the device. The key factor affecting the FF of solar cells is the SRH recombination caused by the device’s series resistance, which includes layer and interface resistances. In CZTS devices, the resistive nature of the CZTS/CdS interface resulted in significant recombination and hence reduced the FF [27,53]. The SRH recombination at the heterojunction for both structures are shown in Figure 7. The device with the CdS buffer layer has a high density of carrier recombination at the interface and in the space charge region (SCR), whereas the device with TiO_2_ has a significantly lower density of carrier recombination. These findings explain why the CZTS/TiO_2_ device has a greater FF than the traditional CZTS/CdS structure.

### 3.3. Kesterite/c-Si Tandem Solar Cell Analysis

The findings reported above make the kesterite/TiO_2_ solar cell (>14%) suitable for a tandem configuration with silicon solar cells. Nevertheless, to investigate the impact of the suggested buffer layer on the kesterite/c-Si tandem device, it is important to examine the tandem devices using kesterite/CdS and kesterite/TiO_2_ top subcell structures. In the practical use of the monolithic kesterite/c-Si device, the top and bottom subcells are connected through a tunnel recombination junction. At this junction, the photogenerated holes collected from the back of the top subcell recombine with photogenerated electrons collected from the front of the bottom subcell [54]. For the best functioning of the tandem device, equal amounts of electrons and holes should be collected by each subcell. Splitting the sun’s spectrum by adjusting the bandgap and the thickness of the top subcell is needed to achieve the current matching conditions. First, the single junction kesterite top subcell with various bandgap materials (CZTSSe, CZTS, and CZTGS) with their corresponding optical properties and electron affinities is simulated; to our knowledge, no detailed study has been performed on how kesterite electron affinity varies with the bandgap. As a result, Equation (8) is used to estimate the electron affinities [55]; further information on this equation can be found in [56].

χs = 4.74 − 0.43Eg(4)

**Figure 8:**
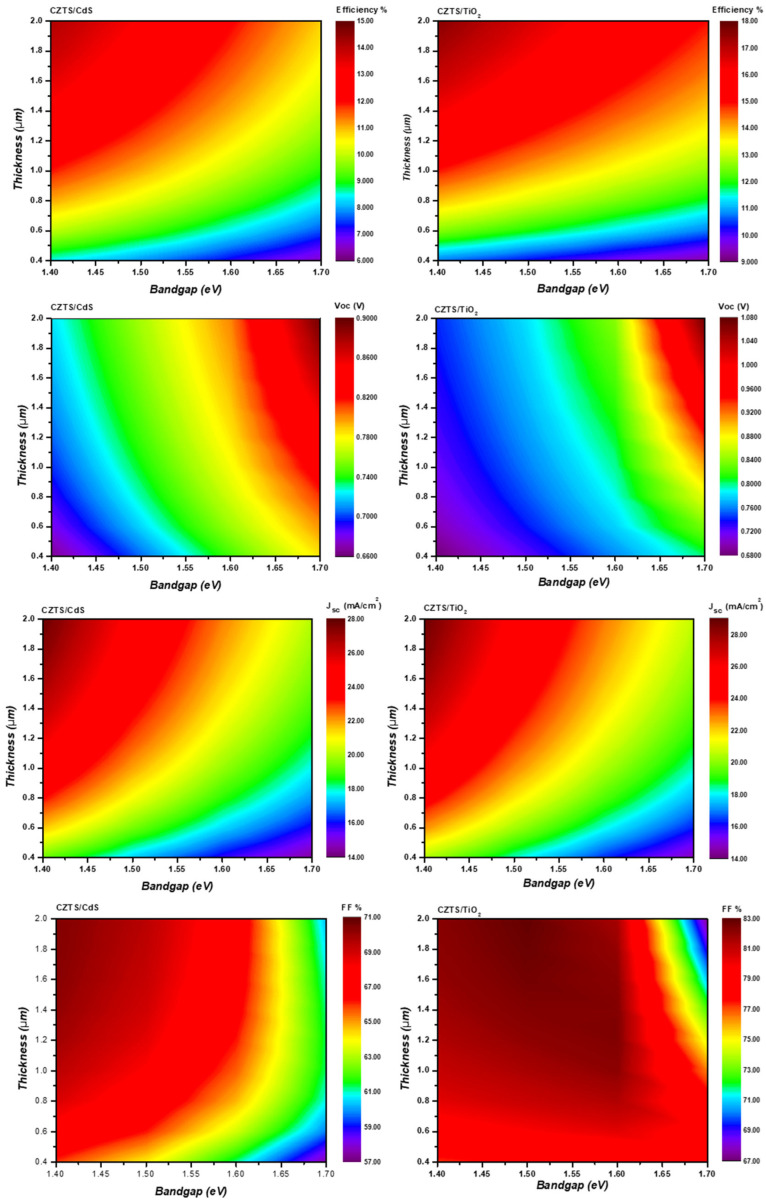
Metric parameters of the top kesterite subcells as a function of the absorber thickness for various bandgap absorbers.

Figure 8 displays the metric parameters of both the kesterite/CdS and kesterite/TiO_2_ subcells under AM1.5 illumination (with different absorber materials) for various absorber thicknesses. In practical development, it is difficult to maintain the properties of kesterite solar cells with thinner absorbers due to incomplete light absorption and the increasing recombination of excess carriers at the back contact [36]. However, the simulation is performed for thicknesses in the range of 0.4–2 µm. As expected, the J_SC_ of both structures are higher for smaller bandgaps. On the other hand, the FF for the kesterite/CdS configuration was found to decrease when the bandgap was higher. This might be due to the enhanced SHR recombination resulting from the modification of the band alignment. In contrast, the FF of the kesterite/TiO_2_ heterostructure shows the opposite behavior; it increases when the bandgap is enlarged, suggesting that TiO_2_ and large bandgap kesterite are well-aligned. The findings are highly encouraging for the future development of large bandgap kesterite devices employing the TiO_2_ buffer layer. J_SC_, V_OC_, and efficiency follow similar trends for both devices and are improved by improving the absorber layer thickness. However, a different behavior of the FF is found in each structure; the FF of kesterite/CdS starts decreasing at a lower thickness (lower than 0.6 µm) when the bandgap is higher than 1.5 eV. However, the FF of the kesterite/TiO_2_ structure is not strongly affected by the variation in the thickness, and a small decrease is observed for higher thicknesses when the bandgap is approximately 1.7 eV. This demonstrated that a thicker kesterite absorber results in a large series resistance, leading to a decrease in the device FF [32].

**Figure 9:**
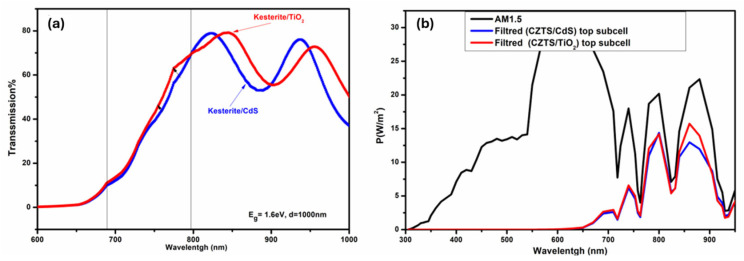
(**a**) Transmission curve of kesterite solar top subcell (Eg = 1.6 eV, d = 1 µm) with TiO_2_ and CdS buffer layer; (**b**) AM1.5 and filtered spectrum from top subcell with TiO_2_ and CdS buffer layers.

To split the spectrum transmitted toward the c-Si bottom subcell, the kesterite top subcell (ITO/MoO_3_/kesterite/CdS or TiO_2_/ZnO/ITO) with varied thicknesses and absorber materials was optically modeled using the TMM method. Figure 9a. depicts the transmission curves of the top subcell for CZTS (Eg = 1.6 eV) and the light transmitted toward the c-Si bottom subcell with CdS and TiO_2_ buffer layers. The transmission value is between 0.4 and 0.8, with interference fringes. The use of wider bandgap TiO_2_ materials improves transmission in the visible region when compared to the CdS buffer layer, and this improves light transmission toward the c-Si bottom subcell, as seen in Figure 9b. These findings support the benefits of utilizing kesterite/TiO_2_ in tandem devices.

**Figure 10:**
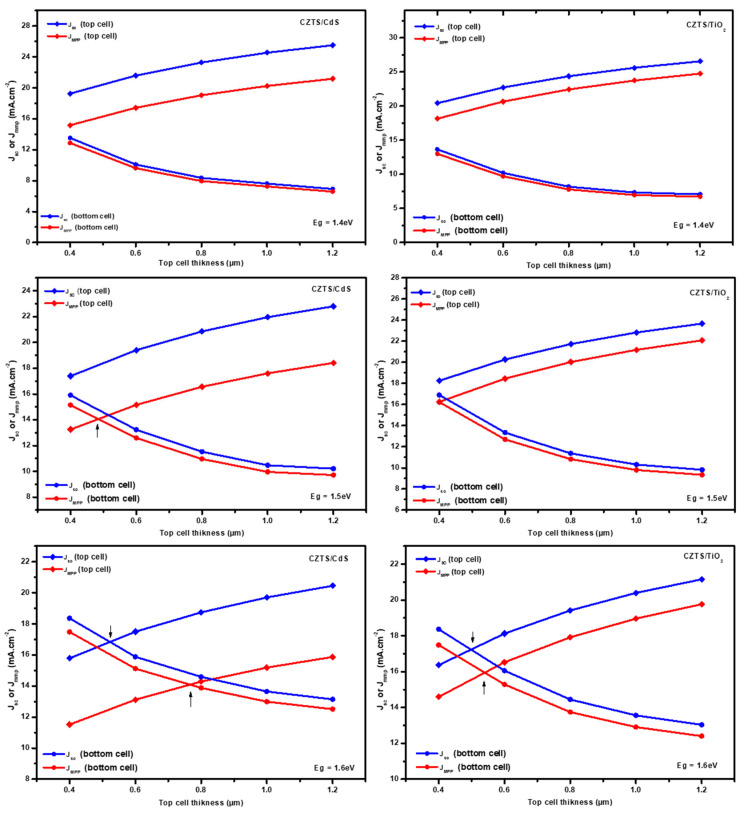
J_MPP_ and J_SC_ of the top and bottom subcells for different top subcell bandgaps and thicknesses.

The current matching conditions under which both subcells generate identical currents were obtained by splitting the solar spectrum. In other words, the transmission spectra are used as a filter in the simulation of the c-Si bottom subcell to attain the current matching conditions. In previous studies, only J_SC_ matching conditions were taken into account in tandem devices [57,58,59]. However, in our study, the kesterite/CdS structure has a lower FF than the c-Si subcell, in which case the J_MPP_ matching conditions are necessary for the best performance of the tandem device [48]. On the other hand, the FF of the simulated kesterite/TiO_2_ configuration is close to the c-Si device FF. Consequently, matching J_SC_ or J_MPP_ will provide similar results. Therefore, both J_SC_ and J_MPP_ matching conditions should be investigated. Figure 10 presents the J_SC_ and J_MPP_ of the silicon bottom subcell and kesterite top subcell for various top subcell absorber material thicknesses and bandgap values. The top subcell’s thickness is limited to 1.2 µm to ensure optimum light transmission to the c-Si bottom subcell. There is no current matching for J_SC_ or J_MPP_ between the two subcells when the top subcell bandgap is 1.4 eV (CZTSSe) for both configurations of the top subcell (kesterite/CdS and kesterite/TiO_2_). The light transmitted toward the bottom subcell is insufficient to produce currents similar to those of the top subcells, which is generally higher for lower bandgap kesterite solar cells. However, because of the lower FF of the kesterite/CdS structure, with a CZTS absorber (Eg = 1.5 eV) the J_MPP_ matching condition was obtained, but no current matching was found with the CZTS/TiO_2_ configuration. With CZTS (Eg = 1.6 eV), both top subcell configurations show two matching conditions, for J_SC_ and for J_MPP_. Furthermore, when the top subcell absorber bandgap takes a value of Eg = 1.7 eV (CZTGS), the light transmitted from the top subcells is adequate to improve the performance of the c-Si bottom subcell, and the kesterite/TiO_2_ subcells show matching conditions for J_SC_ and J_MPP_. However, CZTGS/CdS exhibits only J_SC_ matching conditions due to its smaller FF.

With the present matching conditions acquired, the performance of kesterite/c-Si tandem solar cells were calculated, and the results are given in Table 3. The open circuit voltage of the tandem device is equal to the sum of the open circuit voltages of both subcells, since the top and bottom subcells are series-connected. According to the simulation findings, all calculated efficiencies are greater than those of the kesterite single junction. However, when compared to a single-junction c-Si bottom subcell under AM1.5 illumination, the tandem efficiency with kesterite/CdS as the top subcell is still lower because the kesterite top subcell has lower performance for higher bandgaps and for lower thicknesses. However, all tandem devices simulated with the kesterite/TiO_2_ top subcell exhibit higher metric parameters than the c-Si single junction under AM1.5, which confirms the advantages of the proposed buffer layer for tandem devices. Under J_SC_ matching conditions, the FF of tandem devices with the kesterite/CdS top subcell is low (60%). However, the FF of tandem devices with kesterite/TiO_2_ is greater (of 81%) for both matching conditions (J_SC_ and J_MPP_). These findings prove the advantages of matching J_MPP_ when two subcells with different FFs are combined.

**Figure 11:**
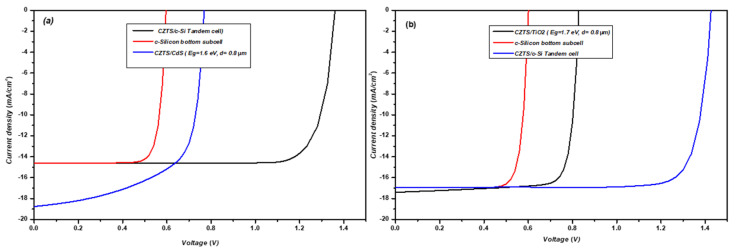
J-V curves of the kesterite top subcell, silicon bottom subcell (under transmitted light from the top subcell), and tandem solar cells with TiO_2_ (**a**) and CdS (**b**) buffer layers.

The photovoltaic parameters of the champion tandem devices with CZTS/CdS and CZTGS/TiO_2_ structures and their subcells are summarized in Table 3. Their J-V characteristics are depicted in Figure 11. The J-V curve of the tandem device is constructed following the assumption that the lower current subcell dominates the J-V characteristics of the tandem device; more details are reported in [48]. Both solar cells achieved the J_MPP_ matching conditions with the c-Si bottom subcell. However, a significant difference in the performance between the devices was observed. The best tandem device using kesterite/CdS as the top subcell shows an efficiency of only 16.50%, with V_OC_ = 1.36 V and J_sc_ = 14.84 mA/cm^2^. The best tandem device with kesterite/TiO_2_ as the top subcell exhibits an efficiency of 20.18%, with V_OC_ = 1.47 V, J_SC_ = 16.95 mA/cm^2^, and a fill factor of 81%. This high improvement in efficiency is due to the large improvement in the performance (e.g., fill factor) of the kesterite/TiO_2_ top subcell, leading to J_MPP_ matching conditions with the silicon bottom subcell for a higher bandgap (1.7 eV) and moderate thickness. At higher absorber bandgaps of the top subcell, the V_OC_ of the tandem is high due to the top subcell having a greater V_OC_. Furthermore, the light transmitted from the top subcell will lead to a better c-Si subcell performance. The results of this simulation show that by inserting a TiO_2_ buffer layer into the kesterite top subcell, the electrical performance of the kesterite/Si tandem could be enhanced. With further advances in kesterite single-junction performance, kesterite/c-Si tandem devices can reach efficiencies similar to those predicted by theoretical studies and will provide an easy path for overcoming the single-junction limit.

**Figure 12:**
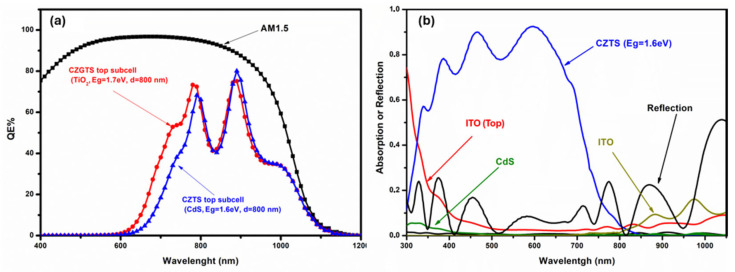
(**a**) QE curves of the silicon bottom subcell (under transmitted light from the top subcell) with TiO_2_ and CdS buffer layers; (**b**) the fraction of light absorbed in each layer and the total reflection of the top subcell.

For the QE curves of the c-Si bottom subcell using the light transmitted from the top subcell with CdS and TiO_2_ at the above matching points, the fraction of light absorbed in each layer of the top subcell (ITO/MoO3/CZTS/CdS/ZnO/n-ZnO) and the total reflection of the device are shown in Figure 12. As can be observed, at higher wavelengths below the bandgap of kesterite material (>750 nm), where the transmitted light should be adequate for a better response of the c-Si bottom subcell, the reflection dominates both the spectra, with values ranging from 20 to 40%, and the absorption of the ITO back electrode, with values ranging from 10 to 20%; this is the reason for the lower QE of the c-Si bottom subcell at this region, as shown in Figure 12b. These results reveal that it is critical to improve the transmission of the top subcell by reducing the reflection in the IR region through optical engineering to have better performance of the tandem device and show that the development of kesterite/c-Si tandem applications may not require well-developed silicon cells as the efficiency of c-Si does not surpass 9% in the best case.

## 4. Conclusions

Optical and electrical simulations of tandem structures with kesterite as the top subcells, using CdS and TiO_2_ as buffer layers and c-Si as the bottom subcell, were examined under different top subcell parameters. The TiO_2_ buffer layer was found to improve large bandgap kesterite performance and show lower interface recombination and parasitic absorption. The tandem devices with kesterite/TiO_2_ simulated under current matching conditions show higher performance than the tandem devices with the kesterite/CdS structure, which is limited by its lower fill factor. The best tandem device with the conventional kesterite/CdS structure (c-Si as the bottom subcell) shows an efficiency of only 16.5%, which is still lower than the c-Si bottom subcell efficiency under AM1.5 illumination (19%). The champion tandem device in our study was obtained with kesterite/TiO_2_, with an absorber bandgap of 1.7 eV (CZTGS) and a thickness of 0.8 µm. This tandem device yields an efficiency of 20.18% with V_OC_ = 1.47 V, J_SC_ = 16.95 mA/cm^2^, and a fill factor of 81%. The findings presented in our study demonstrate the viability of developing highly efficient kesterite/c-Si tandem solar cells using an alternative TiO_2_ buffer layer and indicate that improving the performance of the kesterite top subcell is essential to ensuring the benefit of the tandem device.

## Figures and Tables

**Table 1:** The main material properties used for simulating the tandem cell kesterite/c-Si.

Material Properties	p-Si	n-Si	CZTS	CdS	i-ZnO	ITO	TiO_2_
Thickness (µm)	400	0.5	1.2	0.06	0.05	0.4	0.06
Bandgap (eV)	1.12	1.12	1.5	2.40	3.37	3.37	3.26
Electron affinity (eV)	4.46	4.46	4.1	4.2	4.6	4.6	3.7
Dielectric permittivity	9.1	9.1	7	10	9	9	55
CB (cm^−3^) × 10^19^	2.8	2.8	2.2	2.2	2.2	2.2	2
VB (cm^−3^) × 10^19^	1.040	1.040	1.8	1.8	1.8	1.8	6
Electron mobility (cm^2^/Vs)	1500	1500	100	100	150	150	100
Hole mobility (cm^2^/Vs)	450	450	25	25	25	25	25
N_d_ (cm^−3^)	0	5 × 10^20^	0	10^18^	10^17^	10^20^	10^17^
N_a_ (cm^−3^)	5 × 10^16^	0	1.10^16^	0	0	0	0

**Table 2:** Bulk and interface defect properties used for simulating the baseline solar cells.

Defect Properties	Type	Density (cm^−3^)	Energy Level (eV)	Capture Cross Section (cm^2^)
Bulk CZTS	Single donor	5 × 10^15^	0.900 above Ev	1 × 10^−14^
CdS	Neutral	Grading 1.772 × 10^15^ 9.997 × 10^17^	1.200 above Ev	1 × 10^−13^
CZTS/CdS	Acceptor	2.00 × 10^13^	0.300 above the highest Ev	1.00 × 10^−14^

**Table 3:** Metric parameters of the tandem solar cells simulated at the obtained matching conditions.

Bandgap (eV)	Top Subcell Buffer Layer	Matching Conditions	Thickness (µm)	J_SC_ (mA/cm^2^)	J_MPP_ (mA/cm^2^)	V_OC_ (V)	V_MPP_ (V)	FF %	Eff %
1.5	CdS	J_MPP_	0.50	14.8	14.06	1.31	1.12	80.	15.74
1.6	TiO_2_	J_SC_	0.50	17.26	15.65	1.37	1.19	79	18.62
	TiO_2_	J_MPP_	0.55	16.75	16.00	1.37	1.19	79	19.36
	CdS	J_SC_	0.50	16.86	12.54	1.35	1.15	63	14.42
	CdS	J_MPP_	0.80	14.84	14.11	1.36	1.17	81	16.50
1.7	TiO_2_	J_SC_	0.76	17.18	15.86	1.46	1.25	79	19.82
	TiO_2_	J_MPP_	0.80	16.95	16.14	1.47	1.25	81	20.18
	CdS	J_SC_	0.84	17.16	12.50	1.42	1.20	61.5	15.00

## Data Availability

All data will be shared on request.
